# Medical Students’ Insights on End-of-Life Training and Effective Teaching Strategies

**DOI:** 10.1093/geroni/igaf122.1419

**Published:** 2025-12-31

**Authors:** Cory Bolkan, Katelyn Costanza, Raven Weaver, Anna Roman, Angelique King, Kaela Kyaw, Logan Patterson, Autumn Decker

**Affiliations:** Washington State University, Vancouver, Washington, United States; Washington State University, Spokane, Washington, United States; Washington State University, Pullman, Washington, United States; Washington State University, Spokane, Washington, United States; Washington State University, Spokane, Washington, United States; Washington State University, Spokane, Washington, United States; Washington State University Elson S. Floyd College of Medicine, Spokane, Washington, United States; Rede Group, Pullman, Washington, United States

## Abstract

Medical students typically encounter death during their training, an experience that profoundly shapes their professional aspirations, coping strategies, and personal reflections on mortality. This study explored the emotional impact of these encounters, along with students’ attitudes about death and their experiences with end-of-life (EOL) training. Undergraduate medical students completed an online survey (n = 45) and then a sub-sample (n = 10) participated in in-depth interviews focusing on how EOL encounters or training influenced their professional and personal growth. Using a reflective research methodology, four additional medical student researchers contributed to the narrative analysis of transcribed interviews via an inductive, open coding approach. Over 70% of survey respondents (n = 32) reported both personal and professional experiences with death and 90% wanted more education (i.e., elective course offering). Follow-up interviews highlighted gaps in essential skills like goals-of-care conversations and trauma stewardship. Key themes from the narrative analysis included: (1) developing personal strategies for coping with death, (2) exploring how to comfort others, and (3) conceptualizing what it means to “die well.” Students also emphasized the importance of peer and institutional support to build resilience, manage emotions, navigate EOL care, and prevent burnout. However, students also perceived hesitancy among mentors to discuss EOL options or palliative care, attributing their discomfort to knowledge gaps. These findings highlight the need for comprehensive EOL training in medical curricula to help improve future patient care and to foster resilience, compassion, and well-being in future physicians.

